# Morphology of Aluminum Alloy Foams Produced with Dolomite via Partial Sintering of Precursors

**DOI:** 10.3390/ma12101691

**Published:** 2019-05-24

**Authors:** Ana Maria Medina Ramirez, Ramona Roxana Vintila, Robin A. L. Drew

**Affiliations:** Department of Mechanical Industrial and Aerospace Engineering, Concordia University, Montreal, QC H3G 1M8, Canada; anamaria01@live.com.au (A.M.M.R.); ramona.vintila@mail.mcgill.ca (R.R.V.)

**Keywords:** aluminum foam, dolomite foaming agent, powder metallurgy

## Abstract

Highly expanded, low-cost aluminum-based foams were successfully produced via powder metallurgy using dolomite as foaming agent. Nickel additions (5–15 wt.%) were explored in order to reduce the temperature disparity between dolomite decomposition and the melting range of the metallic matrix. Specific Al–Ni compositions provide appropriate viscosities for effective encapsulation of CO_2_ gas released during dolomite decomposition. A partial sintering step of compacted precursors was introduced prior to foaming, which resulted in high porosity levels (~86%) and significant volume expansion (~250%) in the final product. The partial sintering technique was a key determining factor in obtaining stable, highly expanded cellular structures with homogeneous pores, averaging 3 mm in size and being morphologically comparable with ALPORAS^TM^ foams.

## 1. Introduction

The ever-increasing demand for lower fuel consumption recently triggered exponential research and development of lightweight materials such as aluminum foams [1]. Metallic foams, also known as cellular metals, are usually characterized by a large volume fraction of porosity, sometimes reaching levels above 80% [2]. The excellent impact deformability [3] of aluminum foams, their high energy absorption [4,5,6], and their ability to reduce vibration levels up to 60% make these materials outstanding candidates for crash elements in the automobile industry [7]. In order to fulfill the required mechanical performance, aluminum foams should present appropriate physical characteristics such as being large and uniformly distributed with rounded pores, separated by thin continuous cell walls. The influence of porosity in terms of size, distribution, and morphology on reliability and fatigue performance of manufactured parts is widely recognized in material science, and metallic foams are no exception. For instance, elongated pores constitute failure initiation sites and are detrimental for both the static and dynamic fatigue of components [8]. Moreover, the pore morphology, specifically in metallic foams, directly influences the fracture toughness and energy absorption. In a state-of-the-art study performed by Ahmady et al. [9], it was shown that, for akin manufacturing parameters, the selection of different unit cell geometries results in distinct mechanical behavior, failure mechanisms, and energy absorption values. 

At present, there are various methods to produce aluminum foams [10,11] and each method renders distinct foam characteristics, with variations seen in microstructures, cell morphologies, and relative densities [4]. However, two methods stand out for high-volume mass production at reasonable costs: (i) the melt route (also known as the “direct foaming of melts”), which begins with the metallic matrix in molten state, and (ii) the powder route, a powder metallurgy (PM) process which starts with the matrix in the solid state (metallic powders). There are certain differences in the cellular materials produced via these two methods. For instance, the melt-based process is known to yield higher-porosity structures. Porosity levels of approximately 86% were reported in cellular materials obtained via this method [4]; however, the foams show a greater variation in pore size and cell-wall thickness [4]. The powder-based method presents a net advantage over the melt route due to the nanometric oxide layer (5–15 nm) [12,13] existing on the atomized aluminum particles. It was observed that the oxide layer is broken down during powder compaction and randomly dispersed in the matrix [14,15]. It was also demonstrated that the oxide content of the powders is a contributing factor in foaming behavior and stabilization of the cellular material. During foaming, the oxide dispersion contributes to melt viscosity [14,15] and, thereafter, prevents cell-wall thinning and pore coalescence in the stabilization stage [15,16,17]. 

Both methods of aluminum foam production entail the addition of two agents: (i) the gas source and (ii) the stabilization medium. The gas source can be a gas directly injected into the molten aluminum for the melt-based method and, respectively, foaming agents for the PM route. The foaming agents are customarily metallic hydrides such as TiH_2_, ZrH_2_, or MgH_2_. The stabilization medium can include metallic particles (calcium, aluminum) or non-metallic powders such as ceramics (oxides, carbides, nitrides), intermetallics, fibers, or fly ash. The added particles initially provide optimum viscosity of the melt required for efficient gas retention [4,11,14,18,19,20] and, thereafter, stabilize the cellular materials by preventing drainage and void coalescence in the later foaming stages [15,16,17,18].

Titanium hydrate (TiH_2_) is one of the most widely used blowing agents for aluminum foam production (PM route) due to its effectiveness and low-temperature gas release. Conversely, the low onset decomposition temperature represents also a drawback, owing to the fact that TiH_2_ unaltered (without a diffusion barrier layer) will release gas at temperatures where the aluminum matrix is still in the solid state. This in turn triggers reduced foam expansion and defects in terms of cracks and inhomogeneous pore structure in the final foam. Despite the positive advancements to reduce the temperature mismatch between TiH_2_ gas release and melting temperature of the matrix, either by delaying TiH_2_ decomposition [21,22,23,24] or by depressing the matrix melting range [25], the improvement in synchronizing these two events is still an ongoing effort. Other drawbacks in using TiH_2_ are brought by its elevated price ($80–100/kg), representing up to 25% of the total raw material cost [1] or 10% of the final foam [26], and its hazardous nature, as dust–air mixtures may become explosive. Therefore safer, less expensive foaming agents are highly needed [27,28]. 

A potential blowing agent for aluminum foam production that is both safer and inexpensive is dolomite. In addition to the attractive price ($20–30/ton) and abundance, dolomite, which is a double carbonate of calcium and magnesium (CaMg(CO_3_)_2_), can act simultaneously as a foaming agent and as a foam stabilizer [29]. It can act as a foaming agent owing to the release of CO_2_ gas over a wide temperature range (600–800 °C), and as a stabilizer due to the oxides formed in situ (MgO, CaO) and/or undecomposed CaCO_3_. The theoretical composition by mass of dolomite is 30.41% CaO, 21.86% MgO, and 47.73% CO_2_ [30]. During thermal decomposition, which occurs slowly and subtly (~1 wt.%) at approximately 500 °C followed by a rapid (1.77%/min) and substantial release (~46%) between 590 °C and 785 °C, porous oxides are formed [31]. The oxides act as a scaffold impeding drainage of the molten metal. Moreover, it was reported [32] that, when carbonates are used for aluminum foam production, the reaction between the oxidizing CO_2_ gas released and the molten aluminum results in a thin (<100 nm) oxide film on the cell surface, which contributes significantly to the stability of the cellular structure [32]. 

A survey of the literature reveals, however, that dolomite usage as a blowing agent for aluminum foam production is still not well established. Among the published data, several investigations are prominent. One of the first studies to examine dolomite as a blowing agent was carried out by Papadopoulos et al. [29,33]. The aluminum foams produced from melts presented small (<1 mm), irregular pores, and morphological defects such as big voids and collapsed cells. More homogeneous cell structures were reported by Kevorkijan et al. [5]. The authors used the two methods (the melt route and PM) to fabricate Al foams by adding 5 wt.% SiC stabilizing particles. Although successful via both techniques, the foams resulting from the PM process showed a more uniform microstructure (pore size 0.6–1.3 mm) with well-defined cell borders. In a subsequent study [34], the authors examined the influence of the two foaming agents, TiH_2_ and dolomite, on the morphology of Al–12Si foams via the PM route. Their findings showed that a better stability of the bubbles and a more uniform cell size distribution were achieved when dolomite was used as a foaming agent. Successful results via powder metallurgy were also reported by Koizumi, Gnyloskurenko et al. [35,36,37] in foaming Al–Si–Cu alloys. Although the cell size was relatively small (<1.5 mm), the authors reported an increased cell-wall stability [36] and maximum expansion of dolomite-based foams when compared with foams resulting from individual carbonates [37]. Finally, high expansion levels were obtained by Haesche et al. [38] when foaming AlSi_9_Cu_3_ chips via a thixocasting process. The improvement in expansion levels of dolomite-obtained foams is explained based on the stabilization action of the MgO formed in situ. It was also shown that the stabilization does not occur when CaCO_3_ alone is used as a foaming agent. 

Despite these encouraging findings which report similar/improved microstructures and properties of dolomite-based Al foams when compared to their TiH_2_-based counterparts [5], dolomite is still being used only to a limited extent. It appears that the general agreed upon culprit remains the mismatch between dolomite decomposition temperature and aluminum melting point [29]. Notable efforts [39] were explored recently in order to reduce the temperature interval between these two events. However, the combination of high-temperature treatment, long-term mechanical milling, and acetic acid chemical alteration of dolomite, necessary to lower the gas release temperature [39], presents a limitation at the industrial scale. Therefore, other possible solutions to overcome this limitation need to be considered and explored. In this regard, the addition of an element such as nickel to extend the melting range of the metallic matrix toward the dolomite decomposition interval could present a practical approach. The addition of Ni is expected to have a triple influence on Al foam production. According to the Al–Ni phase diagram [40], two phases of Al + Al_3_Ni coexist for Ni contents between 7 wt.% and 18 wt.% over the temperature interval of dolomite dissociation (i.e., 660 to 800 °C). It is, therefore, anticipated that, in this temperature range, the solid phase provides adequate viscosity in the melt, essential to the foaming process [18]. The second estimated contribution of Ni is the combustion reaction for the synthesis of Al_3_Ni intermetallics reported to increase porosity in the foams. According to Kobashi et al. [41], when Al–Ni specimens are heated to induce the combustion reaction, Al-rich compositions release larger volumes of gas and, consequently, higher-porosity foams are fabricated. Finally, the precipitation of the Al_3_Ni particulates during the rapid cooling can additionally inhibit drainage and stabilize the cellular structure.

The traditional PM technique of aluminum foam production entails, firstly, the fabrication of high-green-density compacts from the metallic powder and blowing agent mixture via warm pressing (~350 °C) [32,42]. This step is generally followed by heating of the compacts to the metallic matrix melting point and blowing agent decomposition temperature (foaming step). However, it was reported that the warm pressing of the powders at such low temperatures is insufficient for achieving uniform cellular structures [43,44]. As a result, additional treatments such as double-axial compaction [45], extrusion, etc. are employed prior to foaming. It was stated that extrusion breaks the tenacious oxide films encasing the metallic powders, creating improved inter-particle bonding. This in turn results in a more uniform microstructure in the final foam. In the present study, rather than using extrusion as an intermediate step, we opted for application of heat alone in the absence of pressure. This relatively low-cost intermediate step, referred to as “partial sintering” is in essence a heat treatment of the compacted precursors to 450 °C for different dwell times. The idea is to promote sufficient inter-particle bonding in the compacts to efficiently encapsulate the released gas and, consequently, to produce highly expanded, homogeneous cellular structures. 

The objectives of the present research were threefold. The first goal was to assess the effectiveness of dolomite as a foaming agent in the fabrication of low-cost aluminum foams (PM method) without the addition of stabilizing particles. The second target was to find a suitable alloying element (Ni) that modifies the properties of the Al matrix in the following manner: (i) increases the matrix melting range to the decomposition interval of dolomite, and (ii) generates a transient semi-solid phase (Al_3_Ni in the melt) to assist pore formation/stability and minimal gas loss at early stages of foaming. Finally, the third objective was to examine the effect of partial sintering on CO_2_ retention, foaming efficiency, and pore morphology. To the best knowledge of the authors, no study exploring the synergetic effect of these three variables was ever conducted.

## 2. Materials and Methods

### 2.1. Precursor Fabrication

Air-atomized aluminum powder (99.5%, −325 mesh; oxygen content of 0.3783 wt.%) and nickel powder (99.7%, 325 mesh; oxygen content of 0.366 wt.%), both obtained from Alfa Aesar, were selected to produce the foam precursors. Four different compositions containing 0%, 5%, 10%, and 15% Ni (by weight) were prepared. Dolomite, with an average particle size of 25 μm, from Fisher Scientific, was used as a foaming agent. The weight percentages of dolomite added to the Al–Ni mixtures were 3 wt.%, 5 wt.%, 7 wt.%, and 10 wt.%. The materials were thoroughly mixed in plastic containers with alumina medium (3:1 ball to powder ratio) in a conventional tumbler mixer for a 1-h blending time. The homogenized powders were thereafter separated from the mixing medium and compacted in a 30-mm-diameter steel die previously lubricated with lithium stearate, and uniaxially cold-pressed at 556 MPa. This was then followed by warm compaction at 350 °C for 60 min and 851 MPa applied pressure until >97% theoretical density was attained for each sample. The obtained precursors are hereinafter referred to “as-compacted”.

### 2.2. Partial Sintering Protocol

Post compaction, some of the specimens were subjected to partial sintering in order to improve the inter-particle bonding. The heat treatment procedure was carried out at 450 °C for 15 or 20 min in air. The resulting precursors are hereinafter referred to as “partially sintered”.

The heat treatment conditions were selected in accordance with the solid-state sintering theory and practice of powder metallurgy [46], in which the sintering temperature is typically selected between 60% and 70% of the melting temperature (*T_m_*) or solidus temperature for single-component metallic powders and binary alloys, respectively. In this compositional range, the solidus temperature for Al–Ni is ~640 °C and, hence, incipient sintering is expected to occur between 385 °C and 448 °C. Although solid-state sintering is sensitive to many factors such as starting particle size, initial density, pore microstructure, heating rate, and atmosphere, when all these parameters are invariable, sintering is directly controlled by the applied temperature. Thereafter, the degree of sintering depends to a large extent on the dwell time at the selected temperature. Complete sintering usually takes 1 to 2 h. The strategy undertaken in this study was selecting the upper end of the sintering temperature (i.e., 450 °C) and short dwell times, i.e., 15 min for partially sintered I specimens and 20 min for partially sintered II precursors. In doing so, we aimed at activating incipient sintering while limiting the formation of the brittle intermetallic compound.

Three-point bending testing was employed to evaluate the sintering level of the precursors. Specimens measuring 30 mm (l) × 15 mm (w) × 4 mm (h) were tested using an MTS Landmark^®^ Testing Solutions machine (MTS Systems Corporation, Eden Prairie, MN, USA) with a span length of 25 mm between two parallel supporting pins. The tests were carried out under a constant 100-kN uniaxial load and a cross-head speed of 0.5 mm/min. The method provides two-fold information on the degree of sintering: (i) the transverse rupture strength values (TRS) give a good indication of the level of inter-particle bonding, and (ii) the resulting fracture surfaces reveal neck development/absence between particles.

### 2.3. Foaming Procedure

The as-compacted and partially sintered specimens were placed in a pre-heated steel crucible previously lubricated with boron nitride powder. The crucible–precursor assembly was thereafter suspended inside an expandometer at 750–800 °C for 16 min. The expandometer, which is in essence a vertical tube furnace, was designed with an exclusive feature that enabled in situ laser displacement measurement of the expanding foam. Additional details of the equipment are provided elsewhere [47].

### 2.4. Characterization Techniques

Several characterization techniques were employed to analyze and evaluate the micro and macro structural features of the starting materials, precursors, and the obtained metallic foams. A scanning electron microscope (SEM, Hitachi–S4300N, Hitachi High-Technologies Corporation, Toronto, ON, Canada) equipped with energy-dispersive X-ray spectroscopy (EDS) was used for morphology and microanalysis. Phase determination was performed with X-ray powder diffraction (XRD; X’Pert Pro-PANalytical, Malvern Panalytical, Montreal, QC, Canada) and confirmed thereafter via EDS. Thermogravimetric analysis (TGA) and differential scanning calorimetry (DSC; Setaram Evolution-24, SETARAM Instrumentation, Caluire, France) were used to determine the decomposition behavior of dolomite. Foam cross-sections and pore size distributions were analyzed via three-dimensional (3D) optical microscopy (Keyence-VHX-2000, KEYENCE Canada Inc., Mississauga, ON, Canada). 

## 3. Results and Discussion

### 3.1. Influence of Partial Sintering of the Precursors on Foaming Evolution

Foaming experiments were carried out with as-compacted specimens and partially sintered precursors. Apart from the heat treatment of the precursors, all foaming conditions such as heating rate, temperature, time, etc. were similar. Also, the compositional range of the additives studied varied simultaneously for both sets of specimens from 3 wt.% to 10 wt.% dolomite and 0 wt.% to 15 wt.% Ni. It was observed that the heat treatment of the precursors has a direct influence on the volume expansion of the final foams. Table 1 shows the average volume expansion for the two categories of precursors with 7 wt.% dolomite and various Ni additions. As seen in Table 1, the as-compacted specimens exhibited only limited foamability, attaining a maximum expansion of approximately 20–30% at higher levels of Ni additions.

In contrast, partially sintered specimens of similar compositions achieved invariably higher expansion levels, ranging between 17% and 244%, depending on the Ni content. This represents 4–9-fold greater expansion levels for foams fabricated from partially sintered precursors when compared to their as-compacted counterparts.

**Figure 1:**
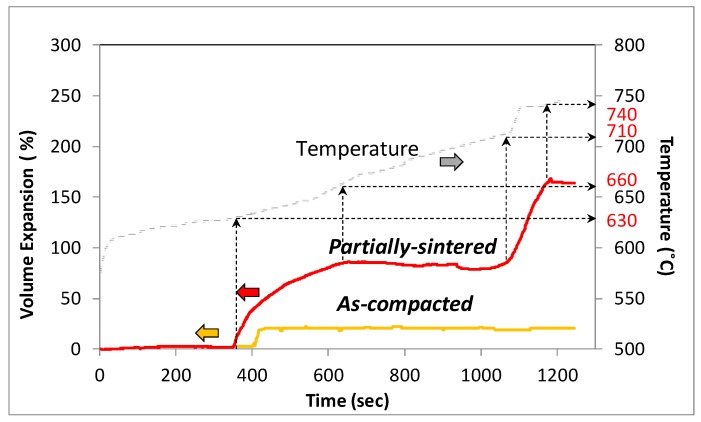
Foaming behavior of Al foam containing 15 wt.% Ni (Al–15Ni) with 10 wt.% dolomite precursors in the as-compacted and partially sintered states.

**Figure 2:**
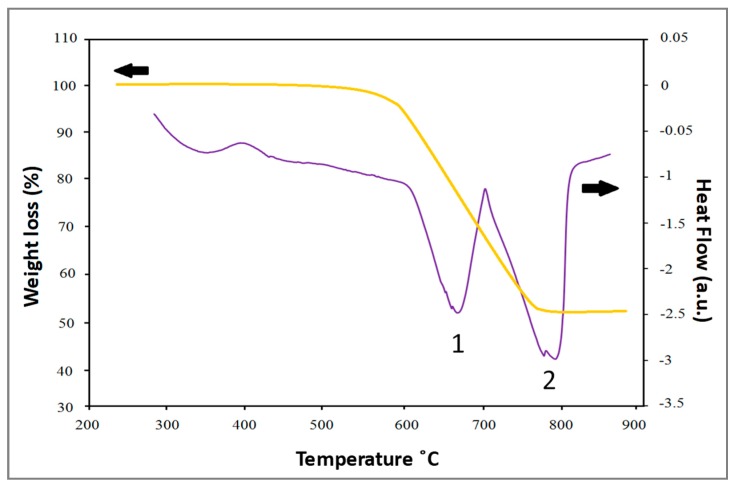
Thermogravimetric analysis (TGA)–differential scanning calorimetry (DSC) curves of dolomite decomposition and weight loss between 200 and 900 °C.

Figure 1 depicts the typical in situ foaming behavior of the as-compacted and partially sintered precursors. As seen in Figure 1, for compositions containing 15 wt.% Ni (Al–15Ni) and 10 wt.% dolomite, the as-compacted specimens exhibited a volume expansion of only 20%, whereas high levels of volume growth of approximately 160% (i.e., eight-fold greater) were recorded for partially sintered samples. This discrepancy in foaming behavior between the as-compacted and partially sintered specimens was observed for all compositions studied. As seen in Figure 1, the partially sintered precursors expanded in two stages. Therefore, it becomes noticeable that the decomposition behavior of dolomite presented in Figure 2 directly influenced the foaming behavior of partially sintered precursors, while it had little or no effect on the as-compacted specimens. As shown by the TGA curve in Figure 2, dolomite decomposition used in this study took place between 590 °C and 780 °C. These findings are in good agreement with experimental data found in the literature [48]. The two endothermic peaks observed on the DSC profile (Figure 2) indicate that the dolomite employed in the current research occurred in two stages. A survey of published data shows a great variability in the one-stage/two-stage decomposition mechanism of dolomite, as it is influenced by dolomite purity (natural/synthetic), experimental conditions, decomposition atmosphere, CO_2_ partial pressure, and particle size [30,49,50]. However, customarily, high-purity, synthetic dolomite decomposes in two stages [51]. In the first step, dolomite decomposes into CaCO_3_, MgO, and CO_2_ [30], followed by CaCO_3_ decomposition into CaO and CO_2_. It was reported that the weight loss in the first stage is approximately 24%, and it is about double in the second stage [30]. By examining the foaming profile of the partially sintered precursors in Figure 1, it can be seen that the expansion indeed followed the two decomposition stages of dolomite shown in Figure 2. The first stage of foam expansion commenced at around 630 °C. The increment in volume was about 80% and occurred rather rapidly within 300 s up to 660 °C. The volume expansion in the specimen in this temperature interval corresponds well with the first endothermic peak on the DSC curve of dolomite dissociation. This stage was followed by a steady state in which the foam expansion arrested for the next 440 s, up to 710 °C. A second, rapid volume increment of 80% occurred between 710 and 740 °C in approximately 100 s. As seen, the foam evolution of the partially sintered precursor is in complete agreement with the two endothermic peaks of dolomite decomposition (Figure 2).

**Figure 3:**
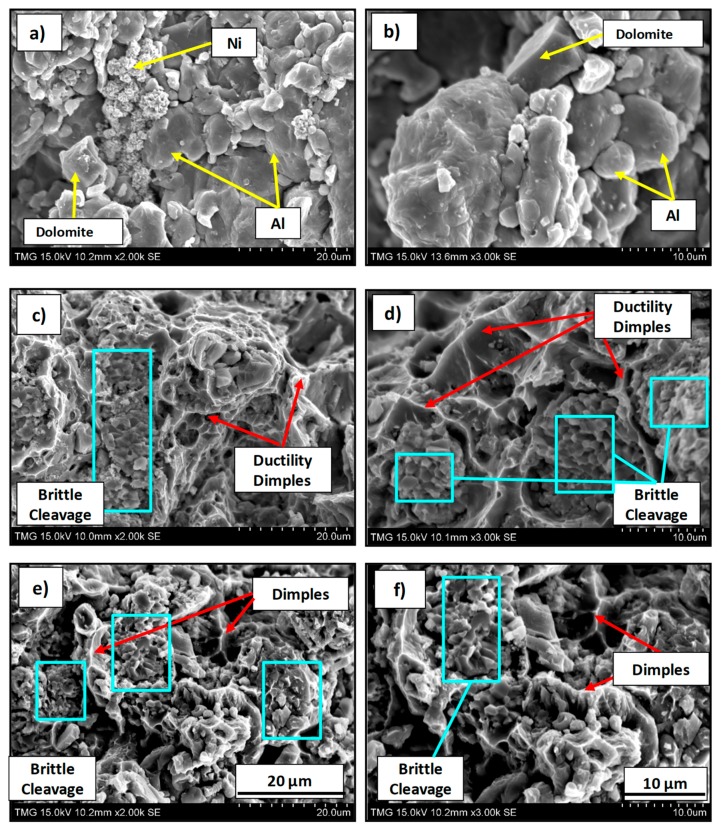
Fracture surface of Al foam containing 10 wt.% Ni (Al–10Ni) with 7 wt.% dolomite compacts with 98% green density: as-compacted (**a**,**b**); partially sintered I at 450 °C, 15 min (**c**,**d**); partially sintered II at 450 °C, 20 min (**e**,**f**).

The as-compacted precursors did not exhibit similar foaming behavior. As depicted in Figure 1, the specimens showed an initial 20% increase in volume at 640 °C, which corresponds with the first endothermic peak of dolomite (Figure 2), and no additional dimensional change was recorded thereafter. The massive CO_2_ escape from the as-compacted specimens is believed to be the cause of such limited expansion. Alternately, the partial sintering of the precursors appeared to efficiently encapsulate the CO_2_ gas owing to the inter-particle bonding developed during the heat treatment process. Knowing that inter-particle connections trigger improved green strength in materials [46], three-point bending tests of precursors were used to evaluate the level of bonding. Details of the testing conditions, TRS values of the precursors, and the volume expansion and porosity level of the final foams are presented in Table 2. Fractographs of the test bars are depicted in Figure 3.

As observed in Table 2, the strength of the as-compacted specimens was 58 MPa, while partially sintered precursors, heat-treated at 450 °C for 15 and 20 min, showed values of 123 MPa and 157 MPa, respectively. The double and triple increase in strength, respectively, indicates that the heat treatment was sufficient to cause surface diffusion and, consequently, a relatively good degree of coherency between particles. Based on the TRS values of the partially sintered precursors, it is estimated that the compacts were in the initial stage of sintering of neck development/incipient neck growth. Direct examination of the resulting fracture surfaces of the test bars (Figure 3) revealed distinct features in the three sets of specimens.

As seen in Figure 3a,b, the fracture surfaces of the as-compacted specimens showed a high degree of particle packing, consistent with their high green density. However, no bonding or interconnectivity was observed between the constituent particles (Al, Ni, and dolomite), which remained constrained within the original boundaries. Alternately, the partially sintered compacts for 15-min and 20-min dwell times (Figure 3c–f) exhibited clear inter-particle bonding. The partially sintered (I and II) specimens showed a combination of ductile dimples and brittle regions. EDS analysis (not presented here) revealed that ductility dimples were aluminum-rich areas, whereas brittle cleavage zones corresponded with the Al_3_Ni phase. The occurrence of dimples in the aluminum matrix and the intergranular fracture mode of the brittle phase confirm the surface diffusion associated with the initial stage of sintering. However, the absence of well-defined particulate borders in these fracture surfaces and the development of the intermetallic phase suggest more a bulk transport mechanism, such as grain boundary diffusion and volume diffusion, consistent with an intermediate sintering stage. Nonetheless, it is evident from the fractographs of the partially sintered I and II precursors that the network of pores, initially interconnected, which were channels for CO_2_ escape in the as-compacted samples, became disrupted and possibly isolated by neck formation and intermetallic phase development during heat treatment.

As expected, longer dwell times (20 min) for partially sintered II precursors generated an increase of the intermetallic phase (Al_3_Ni) with a corresponding increase in the characteristics of brittle fracture (Figure 3d,e). Knowing that the intermetallic phase is harder but more brittle in nature, this increase contributed to the higher strength value (157 MPa) recorded for the partially sintered II specimens (Table 2). Consequently, the strength increment from 123 MPa for partially sintered I specimens to 157 MPa for partially sintered II (Table 2) cannot be straightforwardly attributed to an improvement in sintering. Moreover, an increment in strength in the partially sintered II precursors did not result in higher expansion levels in the foams. This behavior could be explained on the basis of the intermetallic phase brittleness. During foaming, CO_2_ released creates microvoids in the precursor structure, which thereafter coalesce to form a pore. A brittle phase, in which microvoids occur through fast and sudden microcracking, presents a lower encapsulation potential due to the absence of deformability of the ductile phase. It was, therefore, appreciated that the nature (predominantly ductile) and the level of inter-particle bonding (incipient to intermediate sintering) developed during the partial sintering I (450 °C for 15 min) was sufficient for optimum CO_2_ retention.

These findings shed light on the level of inter-particle connectivity required in precursors to develop effective foam expansion. Up until very recently [26], it was believed that achieving the highest green density in the precursors was the sole parameter required for successful foam production. A high green density could be achieved relatively easily in ductile powders, such as aluminum, through cold/hot compaction, extrusion, etc. It is, however, evident that a high green density is not the only factor to be considered. As suitably demonstrated by Lázaro et al. [52] in a study conducted on Al–Si (10 wt.%), in addition to elevated green densities, heat treatments of precursors close to/at sintering temperature of the powders result in a homogeneous structure with absence of cracks, elongated pores, and defects in the final foams. Additionally, according to the same study [52], the higher the heat treatment temperature/dwell time is, the higher the quality of foam will be obtained in terms of pore size consistency and circularity. However, in our case, the upper temperature/dwell time limit was dictated by the increase in the intermetallic phase. Moreover, in the present research the partial sintering protocol was a necessary requirement for effective CO_2_ retention, without which elevated foam expansion could not be achieved.

### 3.2. Influence of Nickel and Dolomite Additions on Foaming Behavior

**Figure 4:**
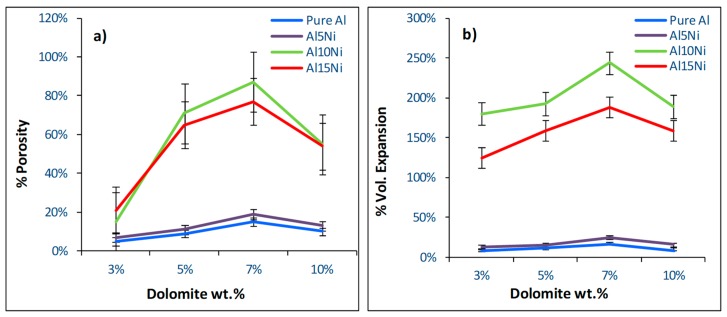
Porosity levels (**a**) and volume expansion (**b**) of foams containing 0 wt.% Ni (pure Al), 5 wt.% Ni (Al–5Ni), 10 wt.% Ni (Al–10Ni), and 15 wt.% Ni (Al–15Ni) as a function of dolomite content.

The present research aimed to determine the optimum combination of dolomite and nickel additions to aluminum in order to obtain the best possible combination of volume expansion, porosity, and homogeneous pore structure in the final product without the addition of stabilizing particles. To this end, an experimental matrix was defined in which the nickel and dolomite contents were varied. All precursors were partially sintered at 450 °C for 15 min. The porosity and volume expansion of the resulting foams are presented in Figure 4a,b, respectively.

#### 3.2.1. Role of Nickel

**Figure 5:**
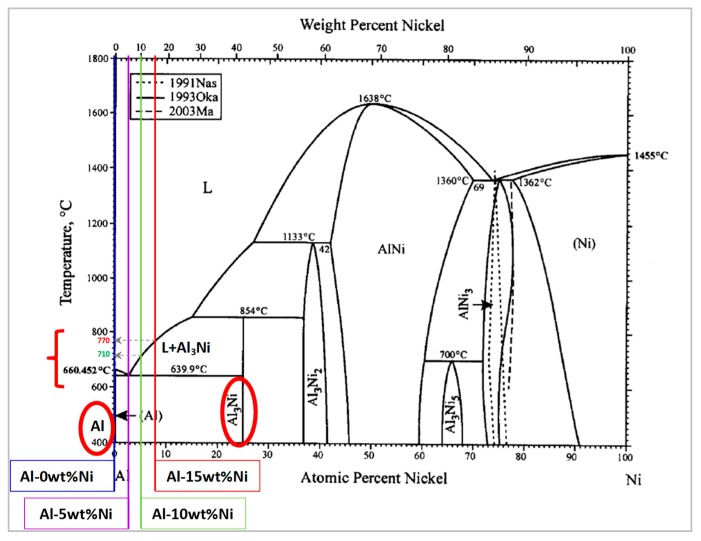
Al–Ni phase diagram showing the Ni additions—adapted from Reference [40].

An important parameter influencing the foaming behavior of the aluminum matrix was the addition of nickel. According to the literature [2], higher porosity levels can be achieved in Al foams by incorporating reinforcement particles or by alloying. Nickel additions of 5, 10, and 15 wt.% were examined. As seen in Figure 4a,b, foams obtained from 0 wt.% Ni (pure Al) and 5 wt.% Ni (Al–5Ni) presented the lowest porosity values and volume expansions. The reduced foamability of samples containing 5 wt.% Ni (Al–5Ni) was related to the fact that Al–5Ni is a eutectic composition which melts at a low and fixed temperature, i.e., 640 °C (Al–Ni phase diagram [40] shown in Figure 5).

**Figure 6:**
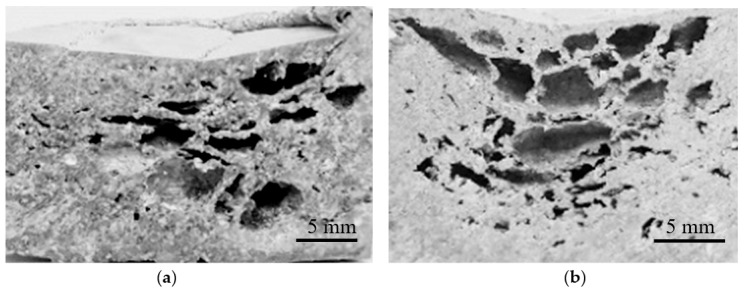
Macrostructures of Al–5Ni foams with 3 wt.% (**a**) and 10 wt.% (**b**) dolomite additions.

The DSC dolomite decomposition (Figure 2) presented two endothermic peaks with a maximum CO_2_ release at approximately 650–660 °C and 770–780 °C. Therefore, at the temperature at which CO_2_ is released, the metallic matrix is entirely in the liquid state with no solid phase, behaving as a thickening agent; hence, any restriction on gas escape imposed by the partial sintering step concludes at the eutectic temperature. As a consequence, the bubbles produced from dolomite decomposition are free to escape at the specimen surface. The reduced foamability and the presence of cracks, elongated pores, and un-foamed areas for both low (3 wt.%) and high (10 wt.%) dolomite additions observed in Al–5Ni samples are depicted in Figure 6a,b, respectively.

**Figure 7:**
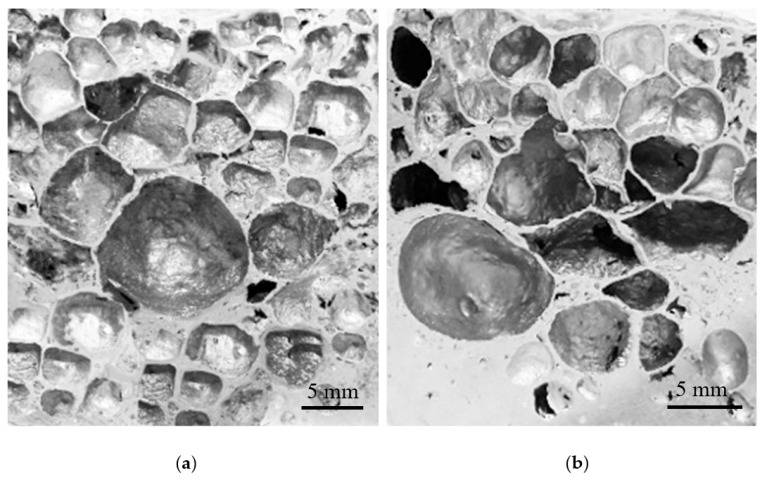
Macrostructures of Al–10Ni foams with 7 wt.% (**a**) and 10 wt.% (**b**) dolomite additions.

Similar behavior was observed for foams produced from pure aluminum, which also enters the liquid state at a fixed temperature (i.e., 660 °C). Despite the melting temperature of Al being higher than Al–5Ni and closer to the first CO_2_ release, the liquid matrix was still unable to retain a considerable amount of foaming gas. These results confirm previous studies [53] which showed that, for stable metallic foams to be successfully produced, an increase in melt viscosity is necessary. This is usually achieved by the presence/incorporation of at least one solid phase in the melt [53]. In this regard, higher Ni-containing compositions, 10 wt.% Ni (Al–10Ni) and 15 wt.% Ni (Al–15Ni), met this requirement. For these compositions, the melting began at the eutectic temperature and it occurred over a wide temperature range: 640 °C to 710 °C for Al–10Ni, and 640 °C to 770 °C for Al–15Ni (Figure 5). Above the eutectic temperature, these compositions contained liquid and solid particles (L + Al_3_Ni), which resulted in an increased viscosity semi-solid. At 650 °C (peak temperature of the first dolomite decomposition), the calculated percentage of solid in Al–10Ni was approximately 10%. Consequently, there existed in the melt a reasonably high amount of solid phase, which raised the viscosity sufficiently to control the CO_2_ released. At the second decomposition stage of dolomite, which commenced at around 710 °C, the system approached the liquidus line. The amount of liquid in the melt increased and only a reduced amount of solid Al_3_Ni particles existed. At this stage, Al_3_Ni particles could provide adequate viscosity for prolonged gas retention and, at the same time, a better dispersion of the blowing gas due to their reduced content. This effect was observed in Al–10Ni foams with 7 wt.% dolomite addition. As depicted in Figure 7a, the resulting foams showed a uniform distribution of porosity with well-defined cell walls and equiaxed spherical pores, which indicates an optimum balance between the internal gas pressure in the developing bubbles and viscosity of the melt.

**Figure 8:**
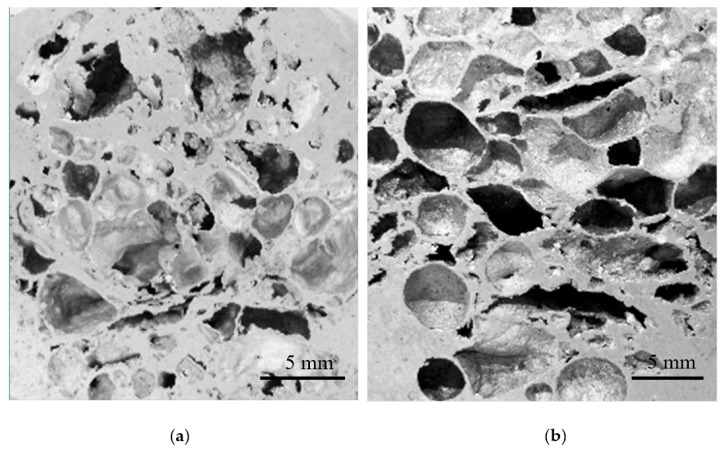
Macrostructures of Al–15Ni foams with 5 wt.% (**a**) and 7 wt.% (**b**) dolomite additions.

Nevertheless, at the same melt viscosity, an increase in foaming agent to 10 wt.% (Al–10Ni) resulted in coalescence of the pores (Figure 7b). The melt was unable to accommodate the higher gas release rate, which consequently triggered rupture of isolated cell walls. For compositions containing 15 wt.% Ni (Al–15Ni), the calculated percentage of solid in the melt at 650 °C was approximately 25% (i.e., 2.5 times higher than in Al–10Ni) and, at 710 °C, was about 15%. This high amount of solid phase in the melt is believed to have excessively raised the melt viscosity, hindering the appropriate foam morphology development. Figure 8a,b depict the macrostructures of Al–15Ni foams, containing 5 wt.% and 7 wt.% foaming agent, respectively.

As observed in Figure 8a, at lower dolomite additions (5 wt.%), the Al–15Ni foams exhibited small, undefined, irregular pores, heterogeneities, cracks, and un-foamed zones. An increase in dolomite content to 7 wt.% (Figure 8b) resulted in an increase in the size of pores, which then became more distinctly spherical but predominately elongated. The presence of cracks was also noted. The morphology of these foams is explained on the basis of increased viscosity generated by the higher Ni content. It is believed that, in both cases, the internal gas pressure of the developing bubbles could not counterbalance the effect of increased viscosity in the melt.

The high amount of solid phase in the melt appeared to hinder the proper gas release in lower dolomite-containing foams (5 wt.%) and the uniform distribution and dispersion of the bubbles, which eventually agglomerated heterogeneously into larger pore clusters in higher dolomite-containing foams (7 wt.%). It can, therefore, be noted that the optimum composition to render maximum volume expansion and uniform distribution of pores is Al–10Ni, which is directly related to an adequate viscosity in the melt.

#### 3.2.2. Role of Dolomite

In order to determine the optimum foaming agent content suitable to maximize volume expansion and render uniform distribution of pores, precursors containing 3, 5, 7, and 10 wt.% dolomite were prepared. As illustrated in Figure 4, an increase in dolomite content prompted an increase in both porosity level and volume expansion up to a concentration of 7 wt.%. However, a further increase to 10 wt.% was not beneficial. The theoretical amount of gas released from samples containing 10 wt.% dolomite is 2.9 × 10^−2^ moles of CO_2_, which is higher than 2.0 × 10^−2^ moles CO_2_ from 7 wt.% dolomite specimens. Nevertheless, this higher gas release was not accompanied by a consequent increase in porosity and volume expansion. This fact is not surprising considering that, for a given melt viscosity, the amount of gas that can be efficiently retained and properly dispersed during foaming is usually the same. More gas released also increases the probability that the gas bubbles will coalesce and create a less uniform porous structure [29], a fact clearly observed in Figure 7b for Al–10Ni foams with 10 wt.% dolomite. These findings are in agreement with the study conducted by Barnhart et al. [17,54], in which it was demonstrated that there is an optimal content of foaming agent above which the maximum expansion of the foams becomes independent.

**Figure 9:**
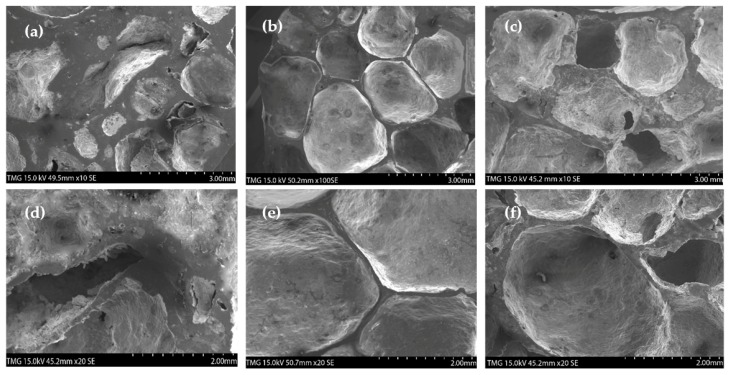
Pore morphologies of Al–10Ni with 5 wt.% dolomite (**a**,**d**), 7 wt.% dolomite (**b**,**e**), and 10 wt.% dolomite (**c**,**f**).

The influence of dolomite additions on porosity development and cell-wall structure is shown in Figure 9 for Al–10Ni compositions.

**Figure 10:**
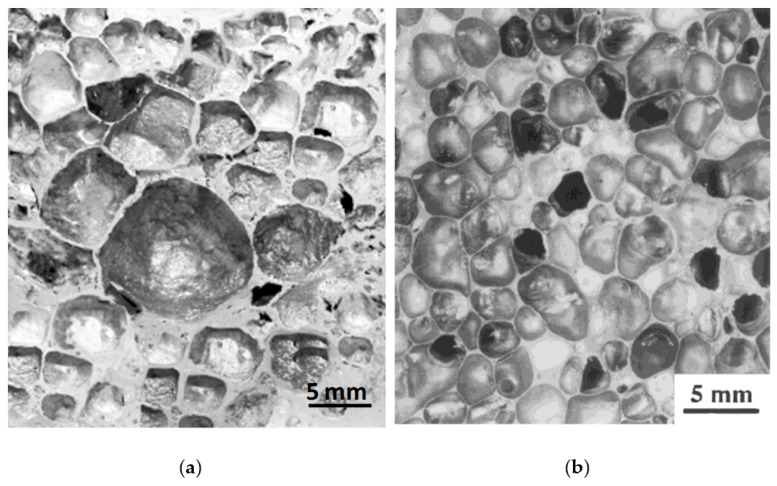
(**a**) Characteristics of Al–10Ni with 7 wt.% dolomite, and (**b**) ALPORAS foams for comparison purposes [20].

The Al–10Ni 5 wt.% dolomite foams (Figure 9a,d—higher magnification) showed irregular, underdeveloped pores and elongated cracks. This indicates that, at this viscosity level, the amount of dolomite was insufficient to generate a well-developed pore structure. The Al–10Ni 10 wt.% dolomite foams (Figure 9c,f—higher magnification) presented ruptures in the cell walls and coalescence of pores. This finding suggests that 10 wt.% dolomite is excessive for effective foaming. However, specimens with a moderate dolomite content of 7 wt.% exhibited a uniform distribution of equiaxed pores. As observed in Figure 9b,e—higher magnification, Al–10Ni with 7 wt.% dolomite foams presented well-developed pores with relatively spherical shape and well-defined cell walls. The measured cell-wall thickness was consistently around 0.23 mm. The average pore size was approximately 3 mm in diameter, which is almost three times larger than the size of pores reported in the literature obtained with the same foaming agent [5,26,29,35,37,38]. Moreover, these cellular materials (Figure 10a) are comparable in terms of morphological characteristics, such as mean cell size (around 3 mm), cell size distribution (in the range of 1–7 mm), well-defined cell walls (consistent thickness), and smoothness of cell surface, with ALPORAS foams (for comparison—Figure 10b), obtained via the melt route using a TiH_2_ gas-generating agent and stabilizing particles [20].

It was noted that, for this combination of additives, the matrix allowed the development of well-formed spherical pores defined by a smooth interior surface (Figure 9e) and absence of defects. Therefore, the foam obtained from partially sintered precursors containing 10 wt.% Ni (Al–10Ni) and 7 wt.% dolomite exhibited an optimum combination of foam porosity, volume expansion, and cell structure homogeneity.

### 3.3. Microstructure, Phase Analysis, and Stabilization Mechanism

The current research entailed fabrication of Al-based foams with dolomite and no addition of external stabilizing agent(s). It was hypothesized that, in the absence of such stabilizing phases, the foaming process and thereafter foam stabilization were assisted by in situ reactions of the constituents and by the contribution of the PM method. Based on the existing research, three stabilization contributions are believed to have occurred in the present study.

The first stabilization contribution in the Al–10Ni with 7 wt.% dolomite should be provided by the oxygen content in the metal powders used for compact fabrication [18]. Both Al and Ni powders were obtained by air atomization and contained approximately 0.38 wt.% and 0.37 wt.% oxygen, respectively. The oxygen content in the powders is adequate to promote foam stability, as it was demonstrated that foams resulting from metallic powders with too low oxygen content (<0.1% O_2_) in N_2_-atomized powders exhibited pronounced drainage [15]. According to studies carried under microgravity [16], the primary role of these oxide particles, also referred to as oxide network particles (ONP [15]) is to prevent bubble coalescence. The stabilization mechanism by ONP is generally agreed upon as being omnipresent in PM foaming of metallic powders obtained by air atomization (or with similar oxygen content). As it is an intrinsic phenomenon occurring during the PM foam process, and as the powders used in the current study meet the oxygen content criteria, it is, therefore, believed that the ONP stabilization mechanism is in effect preventing drainage and cell-wall rupture in the Al–10Ni with 7 wt.% dolomite.

**Figure 11:**
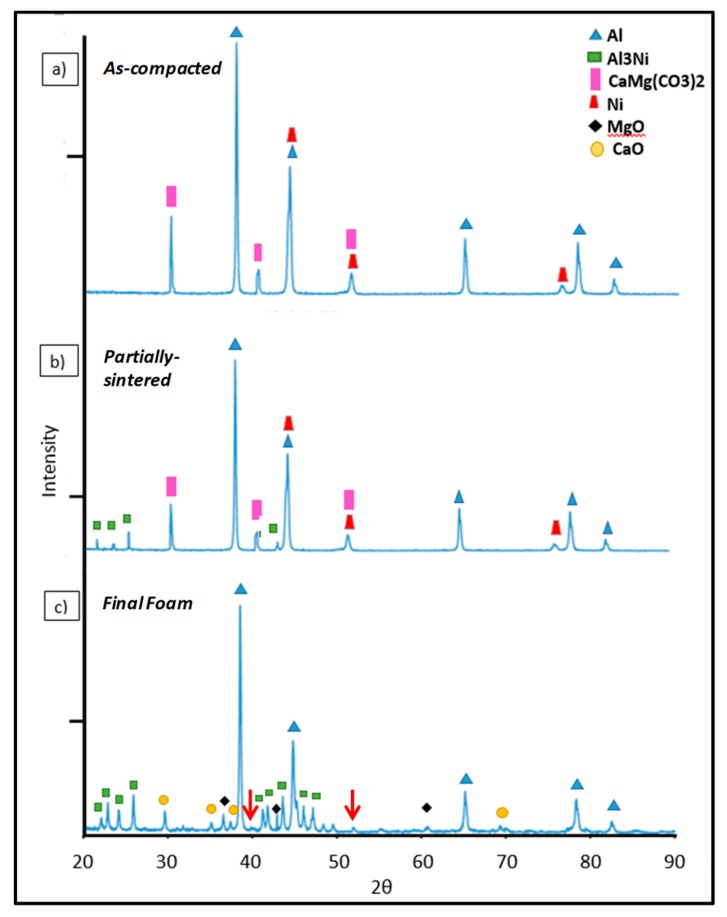
Phase identification in the as-compacted (**a**), partially sintered (**b**), and final foam (**c**) containing Al–10Ni with 7 wt.% dolomite.

**Figure 12:**
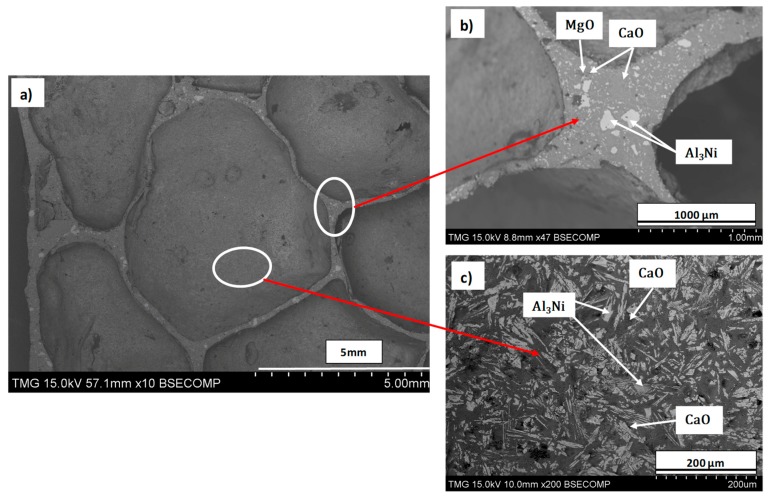
Compositional images of Al–10Ni foams with 7 wt.%: microstructure (**a**), plateau border (**b**), and internal cell surface (**c**).

The second and third stabilization contributions are expected from in situ formed particles during dolomite decomposition (foaming stage) and intermetallic phase development (heat treatment and foam solidification), respectively. In order to evaluate whether these mechanisms are in effect, further analyses were performed. Figure 11 shows the XRD pattern of Al–10Ni with 7 wt.% dolomite in the as-compacted (a), partially sintered precursors (b), and final foams (c). Figure 12 depicts the compositional micrographs of the cellular material obtained (a), identification by EDS, along with characteristic features of the plateau border zones (b), and internal cell surface (c). As observed in Figure 11a, the as-compacted specimens exhibited a combination of Al, Ni, and dolomite. With the heat treatment process (Figure 11b), it is observed that the Al_3_Ni intermetallic phase started to form in the partially sintered precursors. These findings are in agreement with the EDS intermetallic identification in the fracture surface of the partially sintered specimens (Figure 3c,d). The final cellular material (Figure 11c) shows the reflections of the Al matrix, the two oxides (MgO and CaO) resulting from dolomite decomposition, and the Al_3_Ni intermetallic phase. Previous studies [32] showed that, in carbonate-based Al foams, a reaction occurs between molten Al and the CO_2_ gas, which leads to a continuous aluminum oxide film on the internal cell surface.

The oxide layer formed contributes to foam stabilization, preventing cell coalescence and drainage [32,55]. The occurrence of an aluminum oxide layer was also observed in the study conducted by Papadopoulos et al. [29,33] on dolomite-produced Al foams. In the present research, the XRD pattern of the final foams did not conclusively reveal the formation of this oxide layer. However, as seen in Figure 11c, there are several small unidentified peaks (red arrows in Figure 11c) consistent with Al_2_O_3_ reflections. Based on the calculation proposed by Gergely et.al [32], which took into account the foam porosity, the cell size, and foaming temperature, the expected oxide thickness in Al–10Ni with 7 w.% dolomite foams is estimated to be around 40 nm. At this thickness, the oxide layer is not easily detected by XRD. It was shown [32] that the oxide on the internal cell surfaces of carbonate-produced foams presented a smooth continuous layer, constraining the stabilizing particles within the cell walls. This was also observed in the present research. A smooth internal cell surface can be observed in Figure 12a, and particle confinement within the cell wall was detected in the plateau border zones (Figure 12b). It is also possible that the nanometric aluminum oxide layer also developed to some extent due to evolving CO_2_–melt interaction, additionally contributing to foam stabilization.

The two resulting oxides from direct dolomite decomposition (MgO and CaO) were identified in the final foam by both XRD and EDS (Figure 11c and Figure 12). It was reported that, upon dolomite decomposition, the resulting oxides (MgO and CaO) present a porous structure and large surface areas [30,48]. Fine, nanometric MgO particles were observed by Galai et al. [50] deposited on the external surface of the half-decomposed dolomite. These particulate configurations constitute a scaffold for foam stabilization. In fact, the stabilizing effect of MgO against bubble coarsening and coalescence was reported in two independent studies employing dolomite as a foaming agent [29,38]. In addition, CaO identified in our research (Figure 12b,c) is expected to have a similar effect on foam stability, a fact also consistent with studies performed with carbonates as foaming agents [55].

In the current research, a significant improvement in foam homogeneity and stability was brought about by the formation of Al_3_Ni. The two-fold contribution of Al_3_Ni was seen during foam development, as it provided adequate viscosity in the semi-solid state preventing pore coalescence and, thereafter, during solidification, by providing a scaffold for the cellular structure. Homogeneous formation and distribution of Al_3_Ni in the matrix (Figure 12b,c) prevented cell-wall rupture and coalescence, as well as foam collapse and excessive drainage. The formation of Al_3_Ni at 450 °C during the partial sintering is consistent with the phase diagram (Figure 5) and previous research on the in situ formation of Al–Ni intermetallics [56,57,58,59]. A recent study of phase development in the Al–Ni system [59] noted the presence of nanometric Al_3_Ni particles randomly dispersed in the Al matrix after a heat treatment of only 10 min at 610 °C. In the current research, Al_3_Ni precipitates of less than 100 µm formed during solidification, and they were homogeneously dispersed and extensively present in the final cellular material, on both the internal cell surface and within the plateau borders (Figure 12b,c). The effective stabilization mechanism of Al_3_Ni is believed to be similar to the mechanism of endogenous particle stabilization (EPS) first reported by Körner et al. [60] for magnesium foam processing. It appears that these second phases produced in situ act like obstacles in excessive thinning of the cell walls.

The synergetic effect of all the abovementioned contributing factors resulted in a macrostructure of well-developed pores defined by their roundness, smooth surface, absence of defects (Figure 12), and distinct plateau borders in Al–10Ni with 7 wt.% dolomite foams. The foams were fabricated without any additions of stabilization particles, but instead, exclusively by fine-tuning of constituents and processing parameters.

## 4. Conclusions

The fabrication of high-porosity foams with dolomite as a foaming agent and nickel as an alloying element with aluminum was undertaken and successfully demonstrated. This study reveals that dolomite can be an effective, alternative foaming agent to hydrides, providing that the two decomposition stages are matched by a sufficiently viscous liquid phase during foaming. The partial sintering step, introduced here prior to foaming, sealed the dolomite into the microstructure and adequately retained CO_2_ as it was released. This in turn resulted in elevated volume expansions and highly porous structures that closely followed the dolomite decomposition stages.

The optimum combination of foaming agent and alloy content was found to be 7 wt.% dolomite and 10 wt.% Ni, respectively, resulting in approximately 250% volume expansion and 86% final porosity. The foam obtained showed well-developed pores, homogeneously distributed, with relatively spherical shape and consistent cell-wall thickness. The average pore size was 3 mm in diameter, which is almost three times larger than the size of pores reported in the literature with this foaming agent. Furthermore, during foaming, Al_3_Ni, MgO, and CaO were formed at different stages, benefitting foam stabilization.

As demonstrated, the highly porous structures obtained in the present study were the result of (i) a partial-sintering protocol, (ii) selection of the appropriate mass of foaming agent which released the optimum amount of CO_2,_ and (iii) adequate nickel additions that rendered, along with oxide formation, an optimum viscosity in the liquid to efficiently encapsulate, control, and distribute the blowing gas throughout the foaming process. The low-cost fabrication method presented here by fine-tuning of constituents and processing parameters has the potential to bring the large-scale production of Al foams to an economically viable range.

## Figures and Tables

**Table 1:** Average volume expansion of Al foams containing 7 wt.% dolomite resulting from as-compacted and partially sintered precursors for various Ni additions.

	Pure Al	Al-5Ni	Al-10Ni	Al-15Ni
**As-Compacted** Average Vol. Expansion (%)	4	3	30	20
**Partial-Sintered** Average Vol. Expansion (%)	17	25	244	188
**Expansion Factor** (Partial-Sintered/As-Compacted)	~4	~8	~8	~9

**Table 2:** Transverse rupture strength (TRS) data of as-compacted and partial sintered (Al–10Ni) with 7 wt.% dolomite and the characteristics of the resulted foams.

HEAT TREATMENT CONDITIONS			PRECURSOR PROPERTIES	FOAMS CHARACTERISTICS	
Al–10Ni + 7 wt.% Dolomite	Temperature (°C)	Time (min)	TRS (MPa)	Average Vol. Expansion (%)	Average Porosity (%)
As-Compacted	n/a	n/a	58	30	6
Partial-Sintered I	450	15	123	244	77
Partial-Sintered II	450	20	157	243	70
